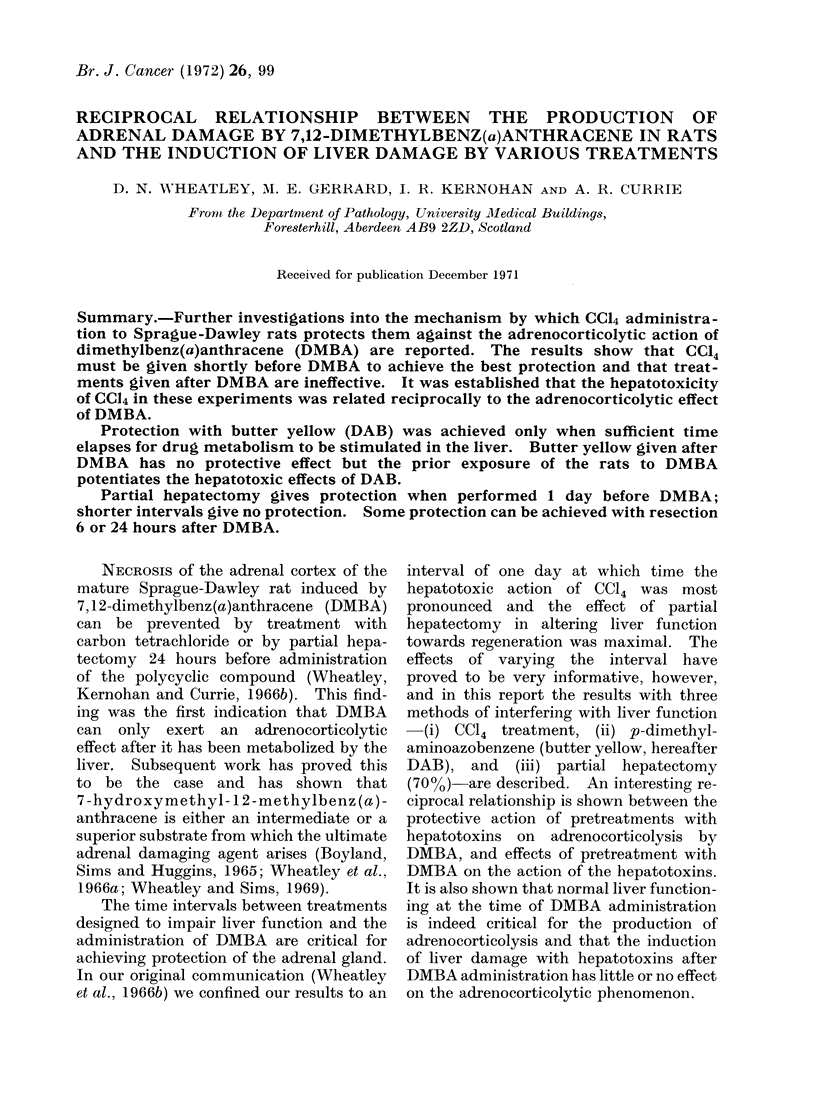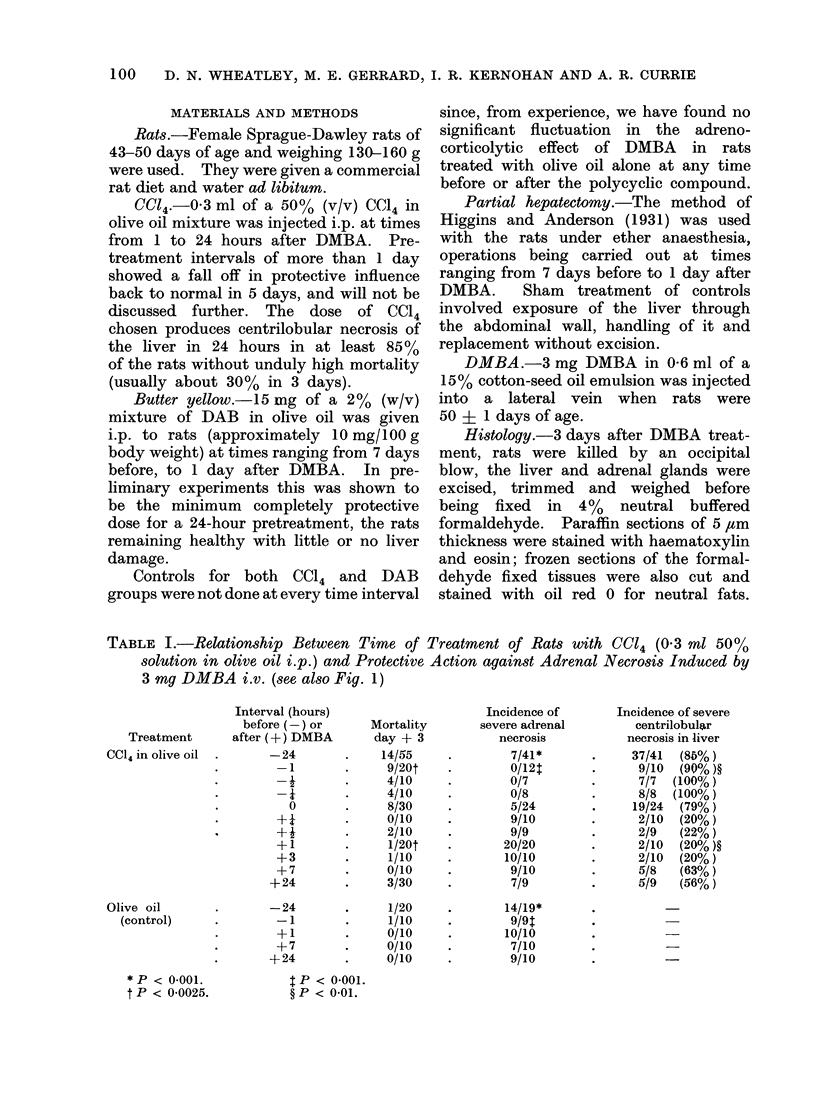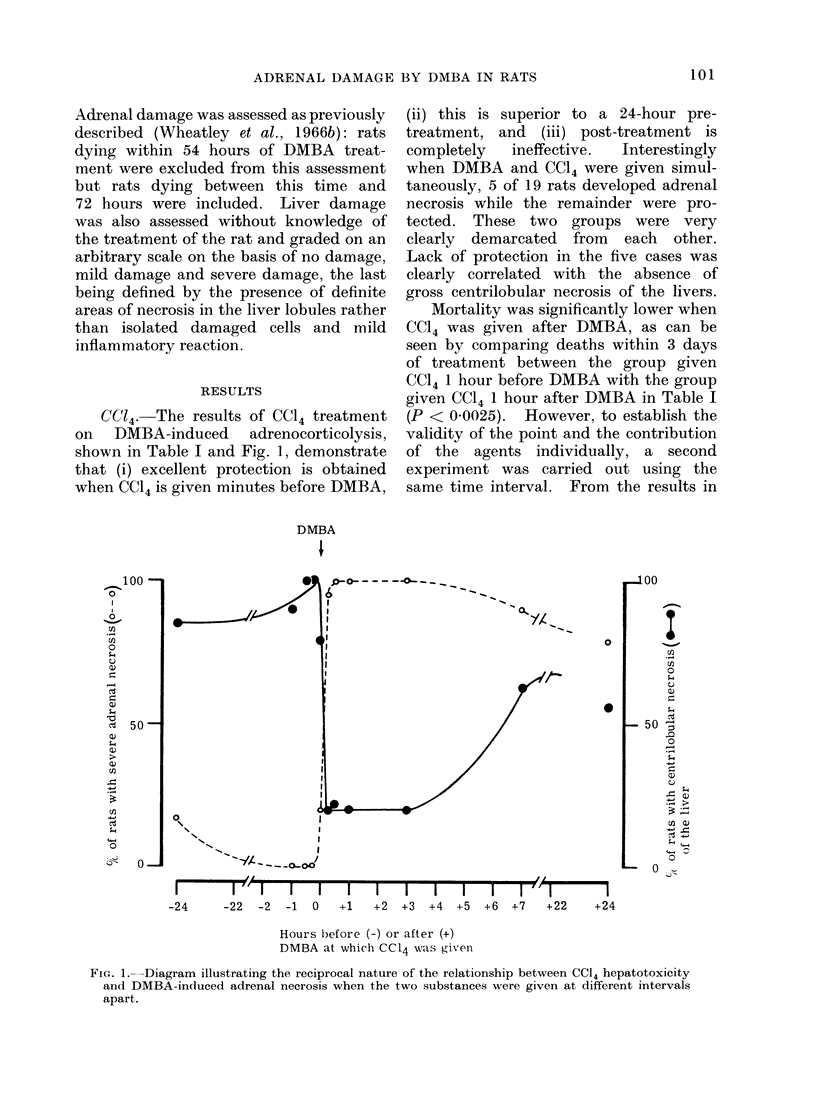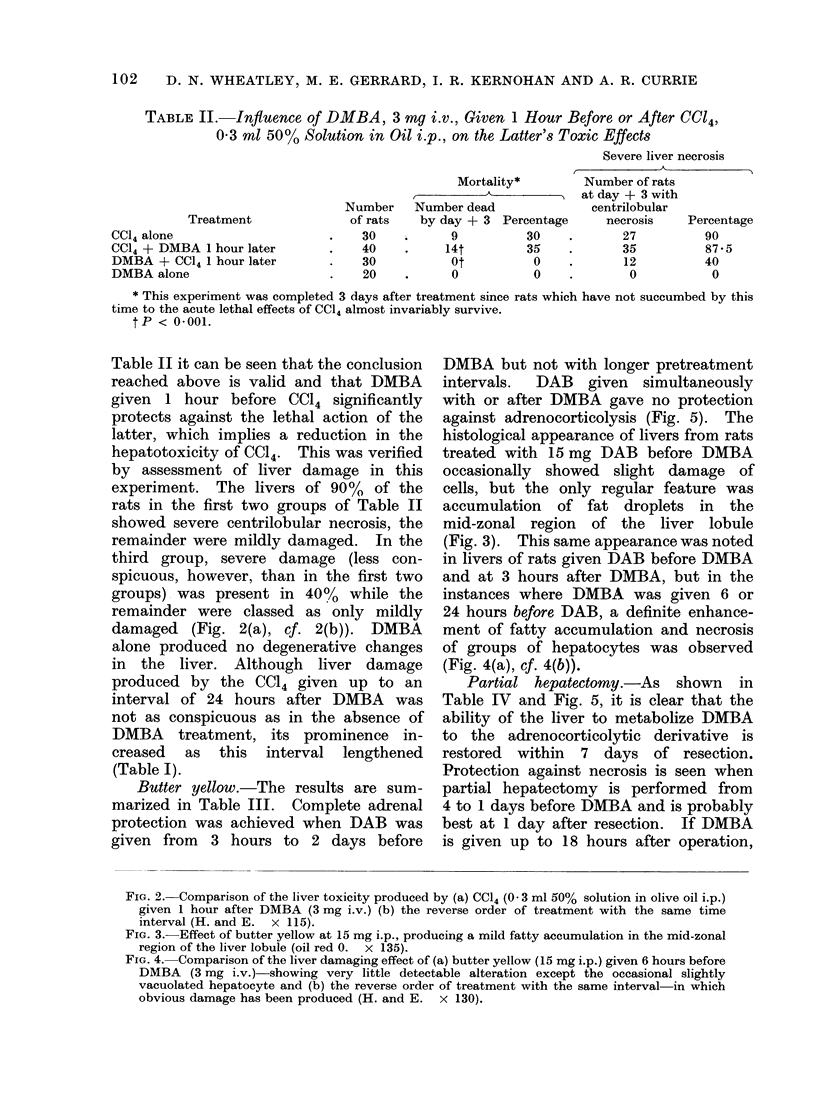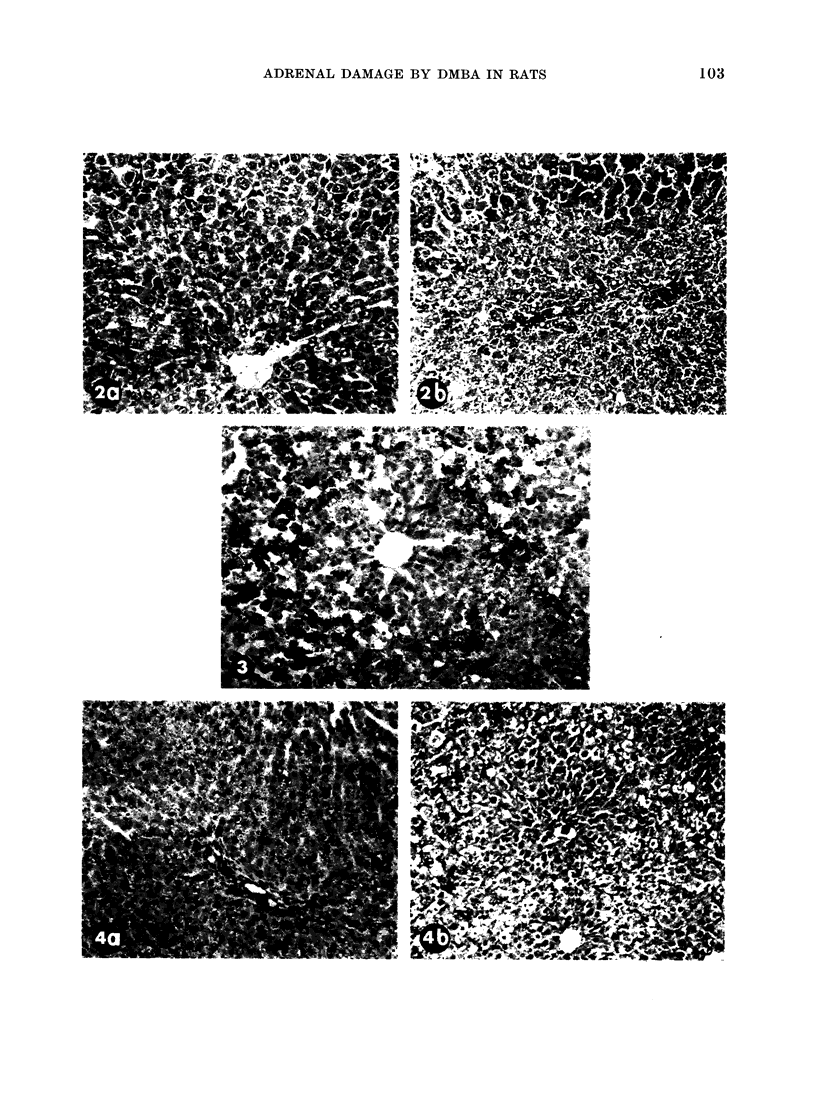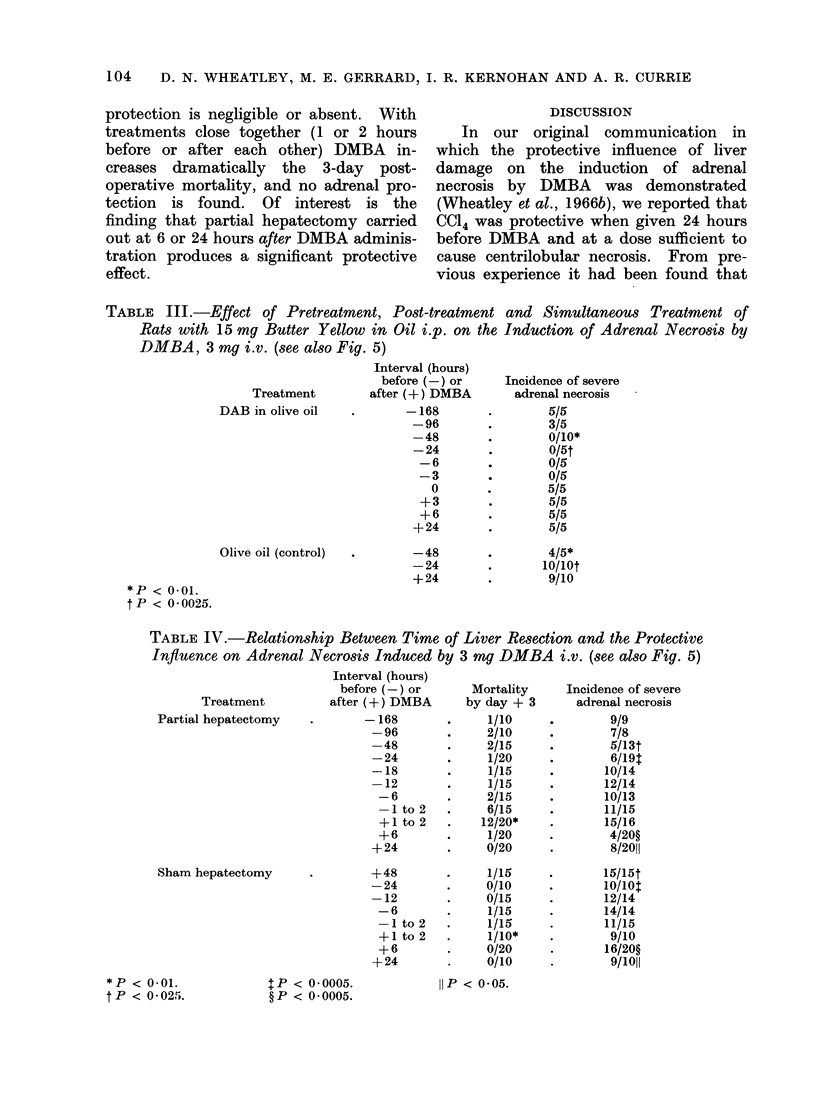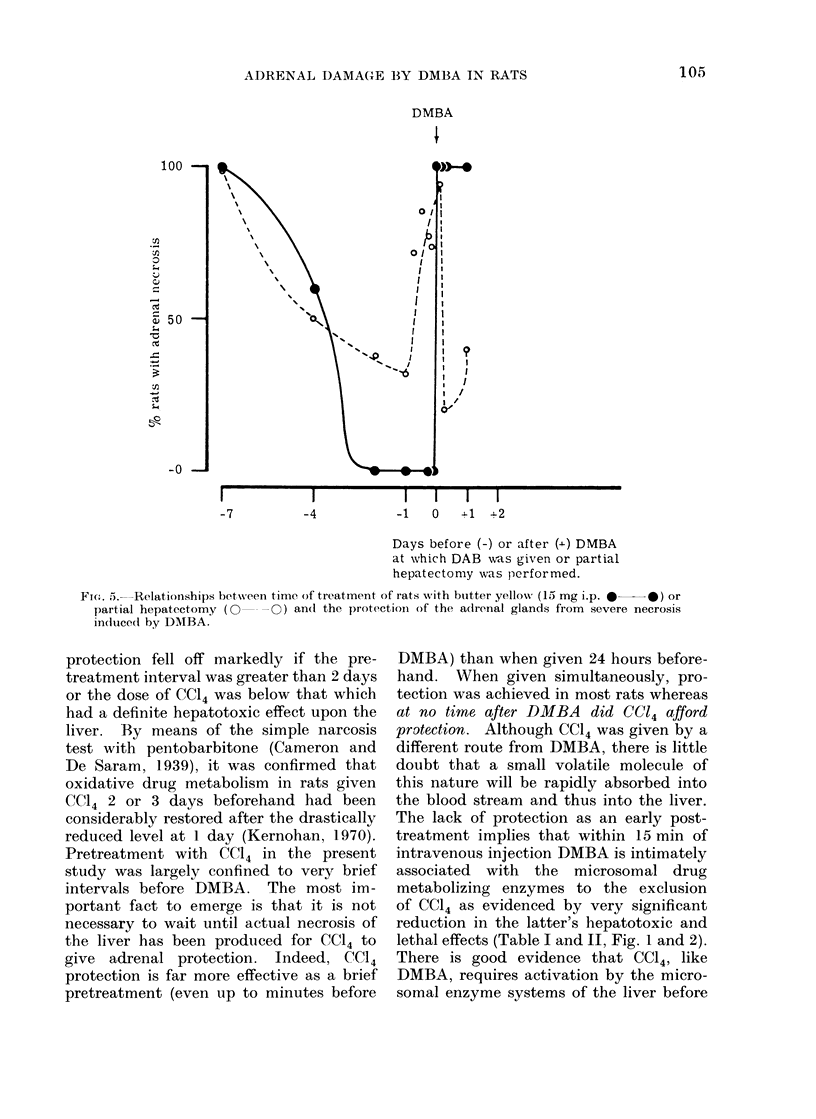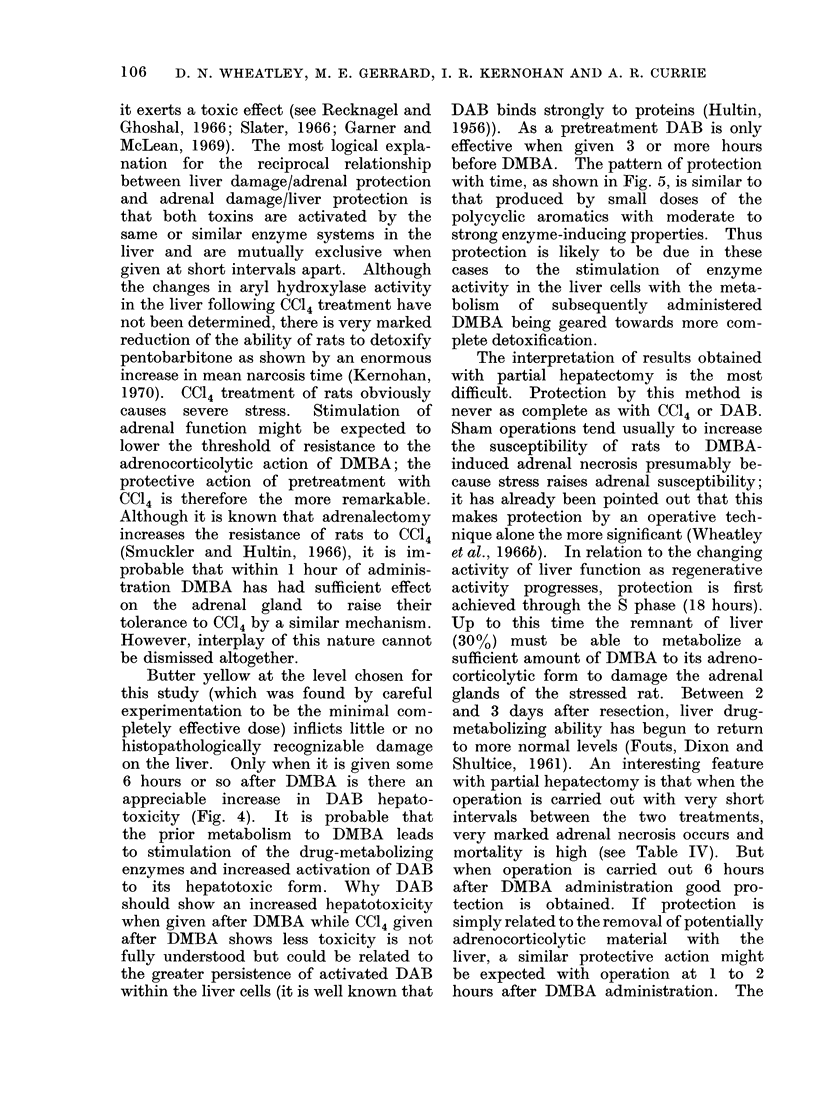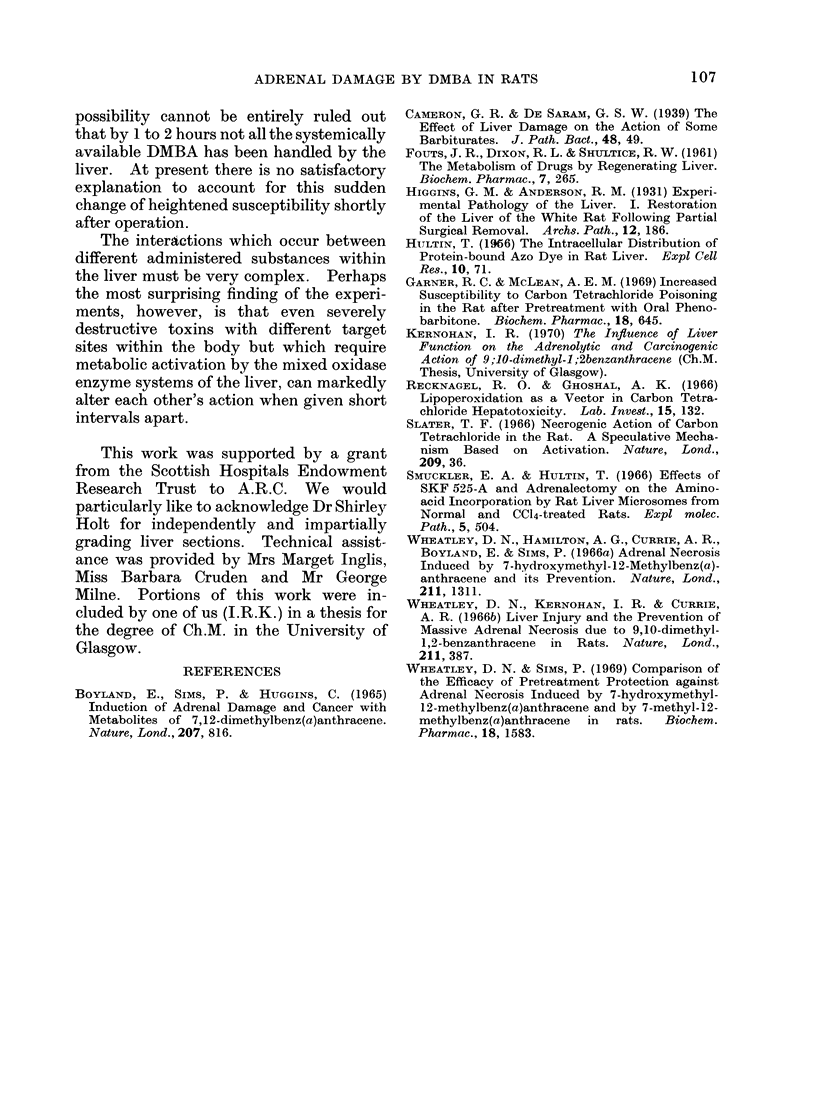# Reciprocal Relationship Between the Production of Adrenal Damage by 7,12-Dimethylbenz(a)anthracene in Rats and the Induction of Liver Damage by Various Treatments

**DOI:** 10.1038/bjc.1972.15

**Published:** 1972-04

**Authors:** D. N. Wheatley, M. E. Gerrard, I. R. Kernohan, A. R. Currie

## Abstract

**Images:**


*Br. J. Cancer* (1972) **26**, 99

### RECIPROCAL RELATIONSHIP BETWEEN THE PRODUCTION OF ADRENAL DAMAGE BY 7,12-DIMETHYLBENZ(*a*)ANTHRACENE IN RATS AND THE INDUCTION OF LIVER DAMAGE BY VARIOUS TREATMENTS

D. N. WHEATLEY, M. E. GERRARD, I. R. KERNOHAN AND A. R. CURRIE

*From the Department of Pathology, University Medical Buildings, Foresterhill, Aberdeen AB9 2ZD, Scotland*

Received for publication December 1971

**Summary.—Further investigations into the mechanism by which $CCl_4$ administration to Sprague-Dawley rats protects them against the adrenocorticolytic action of dimethylbenz(*a*)anthracene (DMBA) are reported. The results show that $CCl_4$ must be given shortly before DMBA to achieve the best protection and that treatments given after DMBA are ineffective. It was established that the hepatotoxicity of $CCl_4$ in these experiments was related reciprocally to the adrenocorticolytic effect of DMBA.**

**Protection with butter yellow (DAB) was achieved only when sufficient time elapses for drug metabolism to be stimulated in the liver. Butter yellow given after DMBA has no protective effect but the prior exposure of the rats to DMBA potentiates the hepatotoxic effects of DAB.**

**Partial hepatectomy gives protection when performed 1 day before DMBA; shorter intervals give no protection. Some protection can be achieved with resection 6 or 24 hours after DMBA.**

NECROSIS of the adrenal cortex of the mature Sprague-Dawley rat induced by 7,12-dimethylbenz(*a*)anthracene (DMBA) can be prevented by treatment with carbon tetrachloride or by partial hepatectomy 24 hours before administration of the polycyclic compound (Wheatley, Kernohan and Currie, 1966*b*). This finding was the first indication that DMBA can only exert an adrenocorticolytic effect after it has been metabolized by the liver. Subsequent work has proved this to be the case and has shown that 7-hydroxymethyl-12-methylbenz(*a*)-anthracene is either an intermediate or a superior substrate from which the ultimate adrenal damaging agent arises (Boyland, Sims and Huggins, 1965; Wheatley *et al.*, 1966*a*; Wheatley and Sims, 1969).

The time intervals between treatments designed to impair liver function and the administration of DMBA are critical for achieving protection of the adrenal gland. In our original communication (Wheatley *et al.*, 1966*b*) we confined our results to an interval of one day at which time the hepatotoxic action of $CCl_4$ was most pronounced and the effect of partial hepatectomy in altering liver function towards regeneration was maximal. The effects of varying the interval have proved to be very informative, however, and in this report the results with three methods of interfering with liver function —(i) $CCl_4$ treatment, (ii) *p*-dimethylaminoazobenzene (butter yellow, hereafter DAB), and (iii) partial hepatectomy (70%)—are described. An interesting reciprocal relationship is shown between the protective action of pretreatments with hepatotoxins on adrenocorticolysis by DMBA, and effects of pretreatment with DMBA on the action of the hepatotoxins. It is also shown that normal liver functioning at the time of DMBA administration is indeed critical for the production of adrenocorticolysis and that the induction of liver damage with hepatotoxins after DMBA administration has little or no effect on the adrenocorticolytic phenomenon.

### MATERIALS AND METHODS

*Rats.*—Female Sprague-Dawley rats of 43–50 days of age and weighing 130–160 g were used. They were given a commercial rat diet and water *ad libitum.*

*$CCl_4$.*—0·3 ml of a 50% (v/v) $CCl_4$ in olive oil mixture was injected i.p. at times from 1 to 24 hours after DMBA. Pretreatment intervals of more than 1 day showed a fall off in protective influence back to normal in 5 days, and will not be discussed further. The dose of $CCl_4$ chosen produces centrilobular necrosis of the liver in 24 hours in at least 85% of the rats without unduly high mortality (usually about 30% in 3 days).

*Butter yellow.*—15 mg of a 2% (w/v) mixture of DAB in olive oil was given i.p. to rats (approximately 10 mg/100 g body weight) at times ranging from 7 days before, to 1 day after DMBA. In preliminary experiments this was shown to be the minimum completely protective dose for a 24-hour pretreatment, the rats remaining healthy with little or no liver damage.

Controls for both $CCl_4$ and DAB groups were not done at every time interval since, from experience, we have found no significant fluctuation in the adrenocorticolytic effect of DMBA in rats treated with olive oil alone at any time before or after the polycyclic compound.

*Partial hepatectomy.*—The method of Higgins and Anderson (1931) was used with the rats under ether anaesthesia, operations being carried out at times ranging from 7 days before to 1 day after DMBA. Sham treatment of controls involved exposure of the liver through the abdominal wall, handling of it and replacement without excision.

*DMBA.*—3 mg DMBA in 0·6 ml of a 15% cotton-seed oil emulsion was injected into a lateral vein when rats were 50 ± 1 days of age.

*Histology.*—3 days after DMBA treatment, rats were killed by an occipital blow, the liver and adrenal glands were excised, trimmed and weighed before being fixed in 4% neutral buffered formaldehyde. Paraffin sections of 5 μm thickness were stained with haematoxylin and eosin; frozen sections of the formaldehyde fixed tissues were also cut and stained with oil red 0 for neutral fats.

TABLE I.—*Relationship Between Time of Treatment of Rats with $CCl_4$ (0·3 ml 50% solution in olive oil i.p.) and Protective Action against Adrenal Necrosis Induced by 3 mg DMBA i.v. (see also Fig. 1)*

| Treatment | Interval (hours) before (−) or after (+) DMBA | Mortality day + 3 | Incidence of severe adrenal necrosis | Incidence of severe centrilobular necrosis in liver |
|---|---|---|---|---|
| $CCl_4$ in olive oil | −24 | 14/55 | 7/41* | 37/41 (85%) |
| | −1 | 9/20† | 0/12‡ | 9/10 (90%)§ |
| | −½ | 4/10 | 0/7 | 7/7 (100%) |
| | −¼ | 4/10 | 0/8 | 8/8 (100%) |
| | 0 | 8/30 | 5/24 | 19/24 (79%) |
| | +¼ | 0/10 | 9/10 | 2/10 (20%) |
| | +½ | 2/10 | 9/9 | 2/9 (22%) |
| | +1 | 1/20† | 20/20 | 2/10 (20%)§ |
| | +3 | 1/10 | 10/10 | 2/10 (20%) |
| | +7 | 0/10 | 9/10 | 5/8 (63%) |
| | +24 | 3/30 | 7/9 | 5/9 (56%) |
| Olive oil (control) | −24 | 1/20 | 14/19* | — |
| | −1 | 1/10 | 9/9‡ | — |
| | +1 | 0/10 | 10/10 | — |
| | +7 | 0/10 | 7/10 | — |
| | +24 | 0/10 | 9/10 | — |

* $P < 0·001$. ‡ $P < 0·001$.
† $P < 0·0025$. § $P < 0·01$.

Adrenal damage was assessed as previously described (Wheatley *et al.*, 1966*b*): rats dying within 54 hours of DMBA treatment were excluded from this assessment but rats dying between this time and 72 hours were included. Liver damage was also assessed without knowledge of the treatment of the rat and graded on an arbitrary scale on the basis of no damage, mild damage and severe damage, the last being defined by the presence of definite areas of necrosis in the liver lobules rather than isolated damaged cells and mild inflammatory reaction.

### RESULTS

*$CCl_4$*.—The results of $CCl_4$ treatment on DMBA-induced adrenocorticolysis, shown in Table I and Fig. 1, demonstrate that (i) excellent protection is obtained when $CCl_4$ is given minutes before DMBA, (ii) this is superior to a 24-hour pretreatment, and (iii) post-treatment is completely ineffective. Interestingly when DMBA and $CCl_4$ were given simultaneously, 5 of 19 rats developed adrenal necrosis while the remainder were protected. These two groups were very clearly demarcated from each other. Lack of protection in the five cases was clearly correlated with the absence of gross centrilobular necrosis of the livers.

Mortality was significantly lower when $CCl_4$ was given after DMBA, as can be seen by comparing deaths within 3 days of treatment between the group given $CCl_4$ 1 hour before DMBA with the group given $CCl_4$ 1 hour after DMBA in Table I ($P < 0{\cdot}0025$). However, to establish the validity of the point and the contribution of the agents individually, a second experiment was carried out using the same time interval. From the results in

FIG. 1.—Diagram illustrating the reciprocal nature of the relationship between $CCl_4$ hepatotoxicity and DMBA-induced adrenal necrosis when the two substances were given at different intervals apart.

TABLE II.—*Influence of DMBA, 3 mg i.v., Given 1 Hour Before or After $CCl_4$, 0·3 ml 50% Solution in Oil i.p., on the Latter's Toxic Effects*

| Treatment | Number of rats | Mortality* Number dead by day + 3 | Mortality* Percentage | Severe liver necrosis Number of rats at day + 3 with centrilobular necrosis | Severe liver necrosis Percentage |
|---|---|---|---|---|---|
| $CCl_4$ alone | 30 | 9 | 30 | 27 | 90 |
| $CCl_4$ + DMBA 1 hour later | 40 | 14† | 35 | 35 | 87·5 |
| DMBA + $CCl_4$ 1 hour later | 30 | 0† | 0 | 12 | 40 |
| DMBA alone | 20 | 0 | 0 | 0 | 0 |

* This experiment was completed 3 days after treatment since rats which have not succumbed by this time to the acute lethal effects of $CCl_4$ almost invariably survive.
† $P < 0·001$.

Table II it can be seen that the conclusion reached above is valid and that DMBA given 1 hour before $CCl_4$ significantly protects against the lethal action of the latter, which implies a reduction in the hepatotoxicity of $CCl_4$. This was verified by assessment of liver damage in this experiment. The livers of 90% of the rats in the first two groups of Table II showed severe centrilobular necrosis, the remainder were mildly damaged. In the third group, severe damage (less conspicuous, however, than in the first two groups) was present in 40% while the remainder were classed as only mildly damaged (Fig. 2(a), *cf.* 2(b)). DMBA alone produced no degenerative changes in the liver. Although liver damage produced by the $CCl_4$ given up to an interval of 24 hours after DMBA was not as conspicuous as in the absence of DMBA treatment, its prominence increased as this interval lengthened (Table I).

*Butter yellow.*—The results are summarized in Table III. Complete adrenal protection was achieved when DAB was given from 3 hours to 2 days before DMBA but not with longer pretreatment intervals. DAB given simultaneously with or after DMBA gave no protection against adrenocorticolysis (Fig. 5). The histological appearance of livers from rats treated with 15 mg DAB before DMBA occasionally showed slight damage of cells, but the only regular feature was accumulation of fat droplets in the mid-zonal region of the liver lobule (Fig. 3). This same appearance was noted in livers of rats given DAB before DMBA and at 3 hours after DMBA, but in the instances where DMBA was given 6 or 24 hours *before* DAB, a definite enhancement of fatty accumulation and necrosis of groups of hepatocytes was observed (Fig. 4(a), *cf.* 4(*b*)).

*Partial hepatectomy.*—As shown in Table IV and Fig. 5, it is clear that the ability of the liver to metabolize DMBA to the adrenocorticolytic derivative is restored within 7 days of resection. Protection against necrosis is seen when partial hepatectomy is performed from 4 to 1 days before DMBA and is probably best at 1 day after resection. If DMBA is given up to 18 hours after operation,

FIG. 2.—Comparison of the liver toxicity produced by (a) $CCl_4$ (0·3 ml 50% solution in olive oil i.p.) given 1 hour after DMBA (3 mg i.v.) (b) the reverse order of treatment with the same time interval (H. and E. × 115).

FIG. 3.—Effect of butter yellow at 15 mg i.p., producing a mild fatty accumulation in the mid-zonal region of the liver lobule (oil red 0. × 135).

FIG. 4.—Comparison of the liver damaging effect of (a) butter yellow (15 mg i.p.) given 6 hours before DMBA (3 mg i.v.)—showing very little detectable alteration except the occasional slightly vacuolated hepatocyte and (b) the reverse order of treatment with the same interval—in which obvious damage has been produced (H. and E. × 130).

2a
2b

3

4a
4b protection is negligible or absent. With treatments close together (1 or 2 hours before or after each other) DMBA increases dramatically the 3-day post-operative mortality, and no adrenal protection is found. Of interest is the finding that partial hepatectomy carried out at 6 or 24 hours *after* DMBA administration produces a significant protective effect.

### DISCUSSION

In our original communication in which the protective influence of liver damage on the induction of adrenal necrosis by DMBA was demonstrated (Wheatley *et al.*, 1966*b*), we reported that $CCl_4$ was protective when given 24 hours before DMBA and at a dose sufficient to cause centrilobular necrosis. From previous experience it had been found that

TABLE III.—*Effect of Pretreatment, Post-treatment and Simultaneous Treatment of Rats with* 15 *mg Butter Yellow in Oil i.p. on the Induction of Adrenal Necrosis by DMBA,* 3 *mg i.v.* (*see also Fig.* 5)

| Treatment | Interval (hours) before (−) or after (+) DMBA | Incidence of severe adrenal necrosis |
|---|---|---|
| DAB in olive oil | −168 | 5/5 |
| | −96 | 3/5 |
| | −48 | 0/10* |
| | −24 | 0/5† |
| | −6 | 0/5 |
| | −3 | 0/5 |
| | 0 | 5/5 |
| | +3 | 5/5 |
| | +6 | 5/5 |
| | +24 | 5/5 |
| Olive oil (control) | −48 | 4/5* |
| | −24 | 10/10† |
| | +24 | 9/10 |

* $P < 0 \cdot 01$.
† $P < 0 \cdot 0025$.

TABLE IV.—*Relationship Between Time of Liver Resection and the Protective Influence on Adrenal Necrosis Induced by* 3 *mg DMBA i.v.* (*see also Fig.* 5)

| Treatment | Interval (hours) before (−) or after (+) DMBA | Mortality by day + 3 | Incidence of severe adrenal necrosis |
|---|---|---|---|
| Partial hepatectomy | −168 | 1/10 | 9/9 |
| | −96 | 2/10 | 7/8 |
| | −48 | 2/15 | 5/13† |
| | −24 | 1/20 | 6/19‡ |
| | −18 | 1/15 | 10/14 |
| | −12 | 1/15 | 12/14 |
| | −6 | 2/15 | 10/13 |
| | −1 to 2 | 6/15 | 11/15 |
| | +1 to 2 | 12/20* | 15/16 |
| | +6 | 1/20 | 4/20§ |
| | +24 | 0/20 | 8/20‖ |
| Sham hepatectomy | +48 | 1/15 | 15/15† |
| | −24 | 0/10 | 10/10‡ |
| | −12 | 0/15 | 12/14 |
| | −6 | 1/15 | 14/14 |
| | −1 to 2 | 1/15 | 11/15 |
| | +1 to 2 | 1/10* | 9/10 |
| | +6 | 0/20 | 16/20§ |
| | +24 | 0/10 | 9/10‖ |

* $P < 0 \cdot 01$. ‡ $P < 0 \cdot 0005$. ‖ $P < 0 \cdot 05$.
† $P < 0 \cdot 025$. § $P < 0 \cdot 0005$.

FIG. 5.—Relationships between time of treatment of rats with butter yellow (15 mg i.p. ●——●) or partial hepatectomy (○- - -○) and the protection of the adrenal glands from severe necrosis induced by DMBA.

protection fell off markedly if the pretreatment interval was greater than 2 days or the dose of $CCl_4$ was below that which had a definite hepatotoxic effect upon the liver. By means of the simple narcosis test with pentobarbitone (Cameron and De Saram, 1939), it was confirmed that oxidative drug metabolism in rats given $CCl_4$ 2 or 3 days beforehand had been considerably restored after the drastically reduced level at 1 day (Kernohan, 1970). Pretreatment with $CCl_4$ in the present study was largely confined to very brief intervals before DMBA. The most important fact to emerge is that it is not necessary to wait until actual necrosis of the liver has been produced for $CCl_4$ to give adrenal protection. Indeed, $CCl_4$ protection is far more effective as a brief pretreatment (even up to minutes before DMBA) than when given 24 hours beforehand. When given simultaneously, protection was achieved in most rats whereas *at no time after DMBA did* $CCl_4$ *afford protection.* Although $CCl_4$ was given by a different route from DMBA, there is little doubt that a small volatile molecule of this nature will be rapidly absorbed into the blood stream and thus into the liver. The lack of protection as an early post-treatment implies that within 15 min of intravenous injection DMBA is intimately associated with the microsomal drug metabolizing enzymes to the exclusion of $CCl_4$ as evidenced by very significant reduction in the latter's hepatotoxic and lethal effects (Table I and II, Fig. 1 and 2). There is good evidence that $CCl_4$, like DMBA, requires activation by the microsomal enzyme systems of the liver before it exerts a toxic effect (see Recknagel and Ghoshal, 1966; Slater, 1966; Garner and McLean, 1969). The most logical explanation for the reciprocal relationship between liver damage/adrenal protection and adrenal damage/liver protection is that both toxins are activated by the same or similar enzyme systems in the liver and are mutually exclusive when given at short intervals apart. Although the changes in aryl hydroxylase activity in the liver following $CCl_4$ treatment have not been determined, there is very marked reduction of the ability of rats to detoxify pentobarbitone as shown by an enormous increase in mean narcosis time (Kernohan, 1970). $CCl_4$ treatment of rats obviously causes severe stress. Stimulation of adrenal function might be expected to lower the threshold of resistance to the adrenocorticolytic action of DMBA; the protective action of pretreatment with $CCl_4$ is therefore the more remarkable. Although it is known that adrenalectomy increases the resistance of rats to $CCl_4$ (Smuckler and Hultin, 1966), it is improbable that within 1 hour of administration DMBA has had sufficient effect on the adrenal gland to raise their tolerance to $CCl_4$ by a similar mechanism. However, interplay of this nature cannot be dismissed altogether.

Butter yellow at the level chosen for this study (which was found by careful experimentation to be the minimal completely effective dose) inflicts little or no histopathologically recognizable damage on the liver. Only when it is given some 6 hours or so after DMBA is there an appreciable increase in DAB hepatotoxicity (Fig. 4). It is probable that the prior metabolism to DMBA leads to stimulation of the drug-metabolizing enzymes and increased activation of DAB to its hepatotoxic form. Why DAB should show an increased hepatotoxicity when given after DMBA while $CCl_4$ given after DMBA shows less toxicity is not fully understood but could be related to the greater persistence of activated DAB within the liver cells (it is well known that DAB binds strongly to proteins (Hultin, 1956)). As a pretreatment DAB is only effective when given 3 or more hours before DMBA. The pattern of protection with time, as shown in Fig. 5, is similar to that produced by small doses of the polycyclic aromatics with moderate to strong enzyme-inducing properties. Thus protection is likely to be due in these cases to the stimulation of enzyme activity in the liver cells with the metabolism of subsequently administered DMBA being geared towards more complete detoxification.

The interpretation of results obtained with partial hepatectomy is the most difficult. Protection by this method is never as complete as with $CCl_4$ or DAB. Sham operations tend usually to increase the susceptibility of rats to DMBA-induced adrenal necrosis presumably because stress raises adrenal susceptibility; it has already been pointed out that this makes protection by an operative technique alone the more significant (Wheatley *et al.*, 1966*b*). In relation to the changing activity of liver function as regenerative activity progresses, protection is first achieved through the S phase (18 hours). Up to this time the remnant of liver (30%) must be able to metabolize a sufficient amount of DMBA to its adrenocorticolytic form to damage the adrenal glands of the stressed rat. Between 2 and 3 days after resection, liver drug-metabolizing ability has begun to return to more normal levels (Fouts, Dixon and Shultice, 1961). An interesting feature with partial hepatectomy is that when the operation is carried out with very short intervals between the two treatments, very marked adrenal necrosis occurs and mortality is high (see Table IV). But when operation is carried out 6 hours after DMBA administration good protection is obtained. If protection is simply related to the removal of potentially adrenocorticolytic material with the liver, a similar protective action might be expected with operation at 1 to 2 hours after DMBA administration. The possibility cannot be entirely ruled out that by 1 to 2 hours not all the systemically available DMBA has been handled by the liver. At present there is no satisfactory explanation to account for this sudden change of heightened susceptibility shortly after operation.

The interactions which occur between different administered substances within the liver must be very complex. Perhaps the most surprising finding of the experiments, however, is that even severely destructive toxins with different target sites within the body but which require metabolic activation by the mixed oxidase enzyme systems of the liver, can markedly alter each other's action when given short intervals apart.

This work was supported by a grant from the Scottish Hospitals Endowment Research Trust to A.R.C. We would particularly like to acknowledge Dr Shirley Holt for independently and impartially grading liver sections. Technical assistance was provided by Mrs Marget Inglis, Miss Barbara Cruden and Mr George Milne. Portions of this work were included by one of us (I.R.K.) in a thesis for the degree of Ch.M. in the University of Glasgow.

### REFERENCES

BOYLAND, E., SIMS, P. & HUGGINS, C. (1965) Induction of Adrenal Damage and Cancer with Metabolites of 7,12-dimethylbenz(*a*)anthracene. *Nature, Lond.*, **207**, 816.

CAMERON, G. R. & DE SARAM, G. S. W. (1939) The Effect of Liver Damage on the Action of Some Barbiturates. *J. Path. Bact.*, **48**, 49.

FOUTS, J. R., DIXON, R. L. & SHULTICE, R. W. (1961) The Metabolism of Drugs by Regenerating Liver. *Biochem. Pharmac.*, **7**, 265.

HIGGINS, G. M. & ANDERSON, R. M. (1931) Experimental Pathology of the Liver. I. Restoration of the Liver of the White Rat Following Partial Surgical Removal. *Archs. Path.*, **12**, 186.

HULTIN, T. (1956) The Intracellular Distribution of Protein-bound Azo Dye in Rat Liver. *Expl Cell Res.*, **10**, 71.

GARNER, R. C. & MCLEAN, A. E. M. (1969) Increased Susceptibility to Carbon Tetrachloride Poisoning in the Rat after Pretreatment with Oral Phenobarbitone. *Biochem. Pharmac.*, **18**, 645.

KERNOHAN, I. R. (1970) *The Influence of Liver Function on the Adrenolytic and Carcinogenic Action of 9;10-dimethyl-1;2benzanthracene* (Ch.M. Thesis, University of Glasgow).

RECKNAGEL, R. O. & GHOSHAL, A. K. (1966) Lipoperoxidation as a Vector in Carbon Tetrachloride Hepatotoxicity. *Lab. Invest.*, **15**, 132.

SLATER, T. F. (1966) Necrogenic Action of Carbon Tetrachloride in the Rat. A Speculative Mechanism Based on Activation. *Nature, Lond.*, **209**, 36.

SMUCKLER, E. A. & HULTIN, T. (1966) Effects of SKF 525-A and Adrenalectomy on the Amino-acid Incorporation by Rat Liver Microsomes from Normal and $CCl_4$-treated Rats. *Expl molec. Path.*, **5**, 504.

WHEATLEY, D. N., HAMILTON, A. G., CURRIE, A. R., BOYLAND, E. & SIMS, P. (1966*a*) Adrenal Necrosis Induced by 7-hydroxymethyl-12-Methylbenz(*a*)-anthracene and its Prevention. *Nature, Lond.*, **211**, 1311.

WHEATLEY, D. N., KERNOHAN, I. R. & CURRIE, A. R. (1966*b*) Liver Injury and the Prevention of Massive Adrenal Necrosis due to 9,10-dimethyl-1,2-benzanthracene in Rats. *Nature, Lond.*, **211**, 387.

WHEATLEY, D. N. & SIMS, P. (1969) Comparison of the Efficacy of Pretreatment Protection against Adrenal Necrosis Induced by 7-hydroxymethyl-12-methylbenz(*a*)anthracene and by 7-methyl-12-methylbenz(*a*)anthracene in rats. *Biochem. Pharmac.*, **18**, 1583.